# Association between walking speed and cognitive domain functions in Chinese suburban-dwelling older adults

**DOI:** 10.3389/fnagi.2022.935291

**Published:** 2022-08-01

**Authors:** Hong Wang, Hui Zhang, Yaoxin Chen, Ming Cai, Cailian Guo, Peijie Chen

**Affiliations:** ^1^School of Kinesiology, Shanghai University of Sport, Shanghai, China; ^2^College of Rehabilitation Sciences, Shanghai University of Medicine and Health Sciences, Shanghai, China; ^3^Jiangwan Hospital of Shanghai Hongkou District, Shanghai University of Medicine and Health Science Affiliated First Rehabilitation Hospital, Shanghai, China

**Keywords:** cognitive impairment, sex-difference, orientation, slow walking speed, cognitive domain functions

## Abstract

**Objective:**

To examine the relationship among walking speed, cognitive impairment, and cognitive domain functions in older men and women living in a Chinese suburban community.

**Methods:**

In total, 625 elderly (72.54 ± 5.80 years old) men (*n* = 258) and women (*n* = 367) from the Chongming district of Shanghai participated in this study. All participants had Mini-Mental State Examination (MMSE), 4-m walking test, medical history questionnaire, and physical examination. They were grouped according to walking speed (>0.8 vs. ≤ 0.8 m/s) with the stratification of sex. The odds ratio (*OR*) and the 95% confidence interval (*CI*) were assessed using the chi-square test and logistic regression analysis.

**Results:**

Around 11.6% of men and 14.2% of women had slow walking speeds. After adjusting for age, body mass index (BMI), education level, spouse, faller, the Geriatric Depression Scale (GDS) score, heart disease, stroke, arthritis, and low back pain, walking speed was negatively related to cognitive impairment in men (*OR* 0.11 [95% *CI*: 0.01, 0.94]; *p* = 0.043). In addition, the relationship between walking speed and impaired orientation was significant in both men (*OR* 0.003 [95% *CI*: 0.001, 0.05]; *p* < 0.001) and women (*OR* 0.15 [95% *CI*: 0.03, 0.75]; *p* = 0.021).

**Conclusion:**

The relationship between walking speed and cognitive impairment was only significant in men, but the association with impaired orientation was found in both men and women. Assessing the walking speed of the elderly is beneficial, which may help with early detection and early therapeutic prevention of cognitive impairment.

## Introduction

Cognitive impairment, such as mild cognitive impairment (MCI) and dementia, is associated with age-related neurodegenerative disease. Impairment of different cognitive domain functions, such as memory, orientation, executive function, and language, may affect daily life to varying degrees (Morley, 2018). In China, the incidence of cognitive impairment among the elderly aged 60 years and above is 12.6–30.0% (Giri et al., 2016; Wu et al., 2019). When the decline in cognitive function progresses to the stage of dementia, neurodegeneration and cognitive impairment will be irreparable. Until then, non-pharmacological interventions, such as exercise, diet, and chronic disease management can help delay or prevent the decline of cognitive function to a certain extent (Rosenberg et al., 2018). Walking has been recognized as one of the most convenient and economical ways of engaging in physical activity. Previous reports have shown links between cognitive function and walking speed in the elderly (Hackett et al., 2018; Liu et al., 2021), but it is unclear whether any specific cognitive domain functions are more strongly associated with walking speed.

Usual walking speeds are chosen by most people for their least energy expenditure and greatest walking efficiency. However, for the elderly, especially those with chronic diseases, walking speed is relatively slow due to limited aerobic capacity and additional demand for energy expenditure associated with physical activity (Schrack et al., 2016). Brain imaging studies have shown that reduced walking speed is associated with smaller hippocampal volume and cerebrocortical mass (Lee et al., 2019). The predictive power of slow walking speed for cognitive impairment has also been demonstrated in older adults (Hackett et al., 2018; Chou et al., 2019; Knapstad et al., 2019). However, studies on the relationship between walking speed and cognitive function in different domains are limited and inconsistent. Some studies reported that walking speed is significantly correlated with all cognitive domain functions (Toots et al., 2019a; Liu et al., 2021). However, other studies have indicated that walking speed is significantly related to the decline of orientation, executive function, and visuospatial function (Zhang et al., 2019; Zuo et al., 2019). Furthermore, walking speed and cognitive function are both reported to decline with age, but sex differences in these declines are still unclear.

Older adults in the suburban community have less opportunity to receive health information and services compared with the urban areas and are more likely to ignore the physical and cognitive problems associated with aging (Liu et al., 2020). The purpose of the present study was to examine the relationship among walking speed, cognitive impairment, and cognitive domain functions in men and women living in a Chinese suburban community to provide a reference for predicting cognitive decline and identifying cognitive impairment early among the population at high risk.

## Methods

### Study design and participants

This was a cross-sectional study conducted from August to November 2018 in the Chongming district of Shanghai, China. The participants of this study were volunteers from the Screening and Rehabilitation Nursing Intervention Study of Cognitive Impairment in the Elderly. All participants were ≥60 years old and lived independently in the community. The exclusion criteria are as follows: (1) those walking with an assistive walking device, such as a cane, walker, or crutch(es); (2) those suffering from serious somatic and/or psychiatric illness, or treatment that may significantly affect walking speed (such as unhealed fractures and tumors); (3) those unable to perform the study required tests (such as 4-m walking test); and (4) those with mental illness, dementia, visual impairment, deafness, or inability to speak that could cause communication difficulties. Finally, 625 participants (258 men (73.16 ± 5.82 years old) and 367 women (72.15 ± 5.73 years old) signed informed consent and were enrolled in the study. The study protocol was approved by the Ethics Committee of the Shanghai University of Medicine and Health Sciences (2020-E4-6200-20-201032-03-210302197009090947). There were training sessions to emphasize the details of the testing and to address the questions for all research associates involved in the study.

### Assessment of cognitive function

Cognitive function was assessed by the Chinese version Mini-Mental State Examination (MMSE), which contains 12 test items to assess several different cognitive domains, such as orientation (0–10), immediate recall (0–3), delayed recall—short-term memory (0–3), attention or concentration ability (0–5), and verbal ability (0–9) (Wu et al., 2019). Higher scores indicate better cognitive function (with a perfect score = 30). Considering the fact that cognitive performance correlates with educational level, the cut-off value of cognitive impairment was adjusted according to the education level, ≤ 17 for illiterate, ≤ 20 for primary school, and ≤ 24 for junior high school and above (Wu et al., 2019; Zhang et al., 2021). The scores for each cognitive domain of the MMSE were transformed into an age-specific *Z*-score for the participants in their 60, 70, and 80s and above, utilizing the data for all participants included in this study. Impairment in each domain of the MMSE was defined as a score >1.0 SDs below the age-specific means (Kondo et al., 2021).

### Assessment of walking speed

Walking speed was measured by a 4-m walking test on a flat, straight corridor. The beginning and end points of the corridor are prominently marked with yellow tape and both points have a 1-m buffer distance (Bohannon and Wang, 2019). The participants were told to walk at their usual pace along the corridor, recording the time in meters per second. They performed the test two times and the mean walking speed (m/s) was calculated and documented (Hooghiemstra et al., 2017). Canes or walkers were allowed to be used. According to walking speed, the participants in this study were divided into two groups: slow walking speed ( ≤ 0.8 m/s) or normal walking speed (>0.8 m/s) as previously described (Ke et al., 2021).

### Covariates

Face-to-face epidemiological questionnaire interviews were performed by trained interviewers. The questionnaire included questions about age, sex, spouse, living alone, education level (0, 1–6, and >6 years), smoking habits, and drinking habits. According to the history of previous falls, participants were divided into the “non-faller” or “faller” groups. When asked the question: “Have you had any falls in the last year?" Participants offering an affirmative answer were assigned to the “faller” group, and others were assigned to the “non-faller” group (Lavedan et al., 2018). In addition, body mass index (BMI) was calculated by dividing weight (kg) by height (m^2^). Physical activity was assessed by the International Physical Activity Questionnaire Short Form (IPAQ-SF) based on the amount of heavy physical activity, moderate physical activity, and walking activity (Zhang et al., 2021). Depressive symptomatology was measured by the 30-item Geriatric Depression Scale (GDS-30), which has a high sensitivity (70.6%) and specificity (70.1%) in a Chinese population sample aged 60 years and over (Rong et al., 2019). Medical or health history was evaluated based on participants' responses (yes or no) to questions about their history, past diagnoses made by physicians, and current or historical medication regimens. Diseases of interest include diabetes mellitus, hypertension, hyperlipidemia, stroke, heart disease, arthritis, and low back pain.

### Statistical analysis

Continuous variables were presented as mean ± standard deviation (SD), continuous non-normal distribution variables, such as IPAQ, were expressed by median and quartile, and classification variables were expressed by percentage (%). Differences in baseline characteristics according to walking speed were analyzed using an independent sample *t*-test, Pearson's chi-square test, and the Mann–Whitney *U*-test. Binary logistic regression models were used to examine the relationship between walking speed and cognitive impairment and impaired cognitive domain function. Adjusted variables in Model 1 included age, BMI, education level, and those in Model 2 included age, BMI, education level, spouse, faller, the GDS score, heart disease, stroke, arthritis, and low back pain. A *p*-value of <0.05 was considered statistically significant. SPSS 25.0 statistical software was used for the analysis.

## Results

A total of 625 participants (258 men and 367 women; average: 72.54 ± 5.80 years old) were included in the analysis. The characteristics of the participants according to sex and categories of walking speed are shown in Table 1. The proportion of slow walking speed was 11.6 and 14.2% in men and women, respectively. Men with slower walking speed were older, had higher GDS scores, lower IPAQ, were more likely to be spouseless, living alone, fallers, and had a higher prevalence of heart disease and cognitive impairment. Women with slower walking speed were older, less educated, had higher GDS scores, lower IPAQ, were more likely to be spouseless, and had a higher prevalence of stroke and cognitive impairment.

**Table 1:** Participant characteristics.

**Variables**	**Men (*n =* 258)**		***P*-value**	**Women (*n =* 367)**		***P*-value**
	**Normal walking speed**	**Slow walking speed**		**Normal walking speed**	**Slow walking speed**	
	**(*n =* 228)**	**(*n =* 30)**		**(*n =* 315)**	**(*n =* 52)**	
Age, yrs	72.75 ± 5.39	76.20 ± 7.85	0.002	71.27 ± 4.80	77.44 ± 7.76	<0.001
BMI, kg/m^2^	25.09 ± 3.37	25.10 ± 4.94	0.995	25.00 ± 3.57	25.73 ± 4.08	0.192
Education, *n* (%), yrs			0.092			<0.001
0	10 (4.4)	4 (13.3)		48 (15.3)	21 (40.3)	
≤ 6	106 (46.5)	15 (50.0)		162 (51.4)	21 (40.3)	
>6	112 (49.1)	11 (36.7)		105 (33.3)	10 (19.2)	
Spouse (%)	214 (94.3)	21 (70.0)	<0.001	241 (76.5)	27 (51.9)	0.001
Living alone (%)	18 (7.9)	5 (16.7)	0.001	49 (15.5)	10 (19.2)	0.419
Smoker (%)	75 (33.0)	7 (23.3)	0.284	3 (1.0)	0 (0)	0.490
Drinker (%)	123 (53.9)	15 (50.0)	0.632	43 (13.7)	7 (13.5)	0.938
Faller (%)	26 (11.4)	9 (30.0)	0.006	54 (17.1)	12 (23.1)	0.262
IPAQ, Met/wk	3,612 (1,017, 9,043)	1,212 (591, 5,662)	0.027	6,132 (2,358, 11,760)	1,593 (486, 6,252)	<0.001
GDS,score	6.11 ± 4.01	7.80 ± 5.32	0.040	7.64 ± 4.80	11.55 ± 6.97	<0.001
**Disease (%)**
Diabetes	31 (13.7)	6 (20.0)	0.352	40 (12.7)	6 (12.2)	0.898
Hypertension	151 (66.5)	22 (73.3)	0.455	176 (56.8)	31 (63.3)	0.393
Hyperlipidemia	34 (15.0)	7 (23.3)	0.240	49 (15.5)	8 (15.3)	0.926
Stroke	23 (10.1)	4 (13.3)	0.591	42 (13.3)	14 (28.6)	0.007
Heart disease	27 (11.8)	10 (33.3)	0.002	56 (17.8)	13 (25.0)	0.162
Arthritis	40 (17.5)	3 (10.3)	0.265	53 (16.8)	14 (26.9)	0.064
Low pack pain	31 (13.7)	6 (20.0)	0.352	70 (22.2)	14 (26.9)	0.357
Cognitive impairment	19 (8.3)	6 (20.0)	0.049	46 (14.6)	15 (28.8)	0.003

*BMI, body mass index; IPAQ, International Physical Activity Questionnaire; GDS, geriatric depression scale*.

Table 2 shows the association between walking speed and cognitive function in men. In Model 1, walking speed was negatively correlated to cognitive impairment (odds ratio [*OR*] 0.12 [95% *CI* 0.02, 0.79]; *p* = 0.024), impaired orientation (*OR* 0.02 [95% *CI* 0.002, 0.15]; *p* < 0.001), and impaired immediate recall (*OR* 0.11 [95% CI 0.02, 0.88]; *p* = 0.031), impaired delayed recall (*OR* 0.27 [95% *CI* 0.07, 0.96]; *p* = 0.045). In Model 2, walking speed remained significantly associated with cognitive impairment (*OR* 0.11 [95% *CI* 0.01, 0.94]; *p* = 0.043) and impaired orientation (*OR* 0.003 [95% *CI* 0.001, 0.05]; *p* < 0.001), while impaired immediate recall (*OR* 0.10 [95% *CI* 0.007, 1.09]; *p* = 0.060), impaired attention, (*OR* 0.43 [95% *CI* 0.06, 3.18]; *p* = 0.400), impaired delayed recall (*OR* 0.33 [95% *CI* 0.08, 1.38]; *p* = 0.130), and impaired language function (*OR* 2.13 [95% *CI* 0.82, 10.78]; *p* = 0.091) were not.

**Table 2:** Associations between walking speed and cognitive impairment and impaired cognitive domain function in men by binary logistic regression analysis.

**Variables**	**Model 1**	***P***	**Model 2**	***P***
	**OR (95%CI)**		**OR (95%CI)**	
Cognitive impairment	0.12 (0.02, 0.79)	0.024*	0.11 (0.01, 0.94)	0.043*
Orientation, impaired	0.02 (0.002, 0.15)	<0.001**	0.003 (0.001, 0.05)	<0.001**
Immediate recall, impaired	0.11 (0.02, 0.88)	0.031*	0.10 (0.007, 1.09)	0.060
Attention, impaired	0.8 (0.16, 4.61)	0.808	0.43 (0.06, 3.18)	0.400
Delayed recall, impaired	0.27 (0.07, 0.96)	0.045*	0.33 (0.08, 1.38)	0.130
Language function, impaired	1.72 (0.70, 5.73)	0.124	2.13 (0.82, 10.78)	0.091

*Model 1: adjusted for age, BMI, education level*.

*Model 2: adjusted for age, BMI, education level, spouse, faller, GDS score, heart disease, stroke, arthritis, low back pain.*

*p <0.05, **p <0.001.

Table 3 shows the association between walking speed and cognitive function in women. After adjusting for age, BMI, education level, spouse, faller, GDS scores, heart disease, stroke, arthritis, and low back pain, walking speed was only negatively related to impaired orientation (*OR* 0.15 [95% *CI* 0.03, 0.75]; *p* = 0.021), while impaired immediate recall (*OR* 0.85 [95% *CI* 0.16, 4.48]; *p* = 0.843), impaired attention, (*OR* 2.00 [95% *CI* 0.45, 4.18]; *p* = 0.362), impaired delayed recall (*OR* 1.33 [95% *CI* 0.35, 5.14]; *p* = 0.672), and impaired language function (*OR* 0.79 [95% *CI* 0.13, 4.76]; *p* = 0.798) were not.

**Table 3:** Associations between walking speed and cognitive impairment and impaired cognitive domain function in women by binary logistic regression analysis.

**Variables**	**Model 1**	***P***	**Model 2**	***P***
	**OR (95%CI)**		**OR (95%CI)**	
Cognitive impairment	0.94 (0.20, 4.55)	0.939	1.42 (0.26, 8.11)	0.689
Orientation, impaired	0.09 (0.02, 0.39)	0.001**	0.15 (0.03, 0.75)	0.021*
Immediate recall, impaired	0.60 (0.13, 2.68)	0.496	0.85 (0.16, 4.48)	0.843
Attention, impaired	1.49 (0.39, 5.79)	0.556	2.00 (0.45, 4.18)	0.362
Delayed recall, impaired	0.69 (0.21, 2.34)	0.551	1.33 (0.35, 5.14)	0.672
Language function, impaired	0.45 (0.09, 2.26)	0.325	0.79 (0.13, 4.76)	0.798

*Model 1: adjusted for age, BMI, education level*.

*Model 2: adjusted for age, BMI, education level, spouse, faller, GDS score, heart disease, stroke, arthritis, low back pain.*

*p <0.05, **p <0.001.

## Discussion

This study explored the relationship between cognitive performance and cognitive domain functions and walking speed in Chinese suburban-dwelling older adults. After adjusting for BMI, educational level, spouse, faller, GDS score and heart disease, stroke, arthritis, and low back pain, we found that slower walking speed was associated with cognitive impairment in men, while the negative association between walking speed and orientation was significant in both women and men.

### Prevalence of cognitive impairment

Previous studies have reported that the prevalence of cognitive impairment ranged from 12.6 to 30% in Chinese community-dwelling older adults with adjusted education-specific cutoffs (Giri et al., 2016; Wu et al., 2019), which was similar to this study (13.8%). In addition, we also observed that the prevalence of cognitive impairment was higher in the elderly with slow walking speed than in those with normal walking speed (25.6 vs. 11.9%, *p* < 0.001), and the prevalence rate was higher in women than in men (16.6 vs. 9.7%, *p* = 0.008).

### Walking speed and cognitive impairment

Walking is the most common exercise method for the elderly living in a suburban setting in China, as it does not require special venues and equipment and is suitable for most of the elderly. Walking speed is a recognized indicator of physical function that gradually decreases with age and can predict a variety of adverse health outcomes, such as physical function, cognitive function, disability, and all-cause death in the elderly (Toots et al., 2019b; Grande et al., 2020). The loss of hippocampal integrity is an important cause of cognitive impairment. Hippocampal atrophy exists in both patients with MCI and Alzheimer's disease (AD), and decreased hippocampal volume can predict a deterioration in cognitive function (Su et al., 2018). Rosso et al. suggested that the relationship between walking speed and cognitive function is supported by a shared neural substrate that includes a smaller right hippocampus (Rosso et al., 2017; Lee et al., 2019).

This study found a negative correlation between walking speed and cognitive impairment, which was in agreement with previous reports that slow walking speed was an independent risk factor for cognitive impairment (Hackett et al., 2018). Compared with the healthy elderly, the elderly with symptoms of cognitive impairment walk slower (Knapstad et al., 2019), which also indicated that the change in walking speed is closely related to the slight decline of cognitive function. Liu et al. reported a significant negative correlation between walking speed and mild cognitive impairment among community elderly people (*OR* 0.25 [95% *CI* 0.10, 0.64]; *p* = 0.004) (Liu et al., 2021). Walking speed is sensitive to changes in cognitive function, so we hypothesized that interventions that increase walking speed may be beneficial to the improvement of cognitive function.

Notably, the decline in physical performance, especially usual walking speed, which decreases with age, also showed sex differences. In this study, the proportion of men who walked slowly was lower than that of women (11.6 vs. 14.2%). Coelho-Junior et al. also reported that the usual walking speed for women rather than men showed an age-dependent decline (Coelho-Junior et al., 2021). Similarly, there are sex differences in the trajectory of cognitive decline, and longitudinal studies show that men perform worse on most cognitive tests adjusted for education level and decline faster than women in terms of episodic memory, language fluency, and verbal ability (Morley, 2018). The results of this study showed that the slower men walked, the more likely they were to have cognitive impairment (*OR* 0.11 [95% *CI* 0.01, 0.94]; *p* = 0.043), however, Tirkkonen et al. reported that the relationship between walking speed and cognitive function did not differ between the sexes among elderly people in the community (Tirkkonen et al., 2021). We speculate that poorer physical performance is likely to indicate worse health, including cognitive function, and more research is needed in the future to investigate whether there are sex differences in the relationship between physical performance and cognitive function.

Previously, it has been reported that exercise intervention improves both physical function and cognitive function (Saez De Asteasu et al., 2019). Both low- and high-intensity physical exercise could significantly improve physical functions, such as balance, walking speed, and endurance in older adults, but the improvement effect of exercise on cognitive function is more obvious in the elderly with better functional ability. For the elderly with dementia, exercise intervention has little effect on cognitive function (Saez De Asteasu et al., 2019; Sanders et al., 2020). Therefore, early diagnosis and early intervention of cognitive impairment are particularly important, the close correlation between cognitive impairment and walking speed can also provide a reference for clinical intervention and public health screening.

### Walking speed and orientation

The detailed division of MMSE domains is important for the evaluation of cognitive impairment. In the process of neuropsychological evaluation, a single cognitive domain function test cannot simultaneously evaluate multiple cognitive domain functions, while the MMSE scale can evaluate multiple cognitive domain functions, such as orientation, immediate recall, and attention, and the results are often applied to the prediction model (Xie et al., 2011).

Although cognitive function gradually declines with age, the function of different cognitive domains may not decline equally at the same time. The decline of MMSE orientation appears in the early stage of cognitive impairment (Xie et al., 2011). In this study, the relationship between walking speed and orientation was significant in both men and women after adjusting for covariates. The brain regions associated with directional force or spatial orientation are concentrated in the retrosplenial cortex (Peer et al., 2015). The retrosplenial cortex is interconnected with the hippocampus and various parahippocampal cortical regions involved in navigation and spatial cognition (Todd and Bucci, 2015), and the cortical loop connects the retrosplenial cortex to M2, the secondary motor cortex (Yamawaki et al., 2016). This may explain the close connection between walking speed and orientation. Liu et al. investigated the correlation between walking speed and cognitive domain function among elderly people living in Chinese communities, and the relationship between walking speed and orientation was also significant, which is consistent with the results of this study (Liu et al., 2021).

### Study limitations and perspectives

This research is the first to focus on the association of walking speed and cognitive impairment in different cognitive domains in men and women in suburban communities in China. In addition, the 4-m walking speed test was easy to practice, suitable for the elderly, and had a good retest reliability (Bohannon and Wang, 2019). The cognitive screening and walking speed tests are also easy to be applied in the community departments of public health.

However, this study also has certain limitations. First, as this study is a cross-sectional study, the impact of changes in walking speed on cognitive function needs to be verified in the elderly with senescence in further studies. Second, the population in this study is the elderly from the Chongming District of Shanghai, and the outcomes and interpretations of the study may not be representative enough of people in other regions or different age groups. The sample size and the number of related areas need to be increased in the future.

## Conclusion

In conclusion, walking speed is an important risk factor for cognitive impairment, independent of certain variables. We observed that the relationship between walking speed and cognitive impairment was only significant in men, but the association with impaired orientation was found in both men and women. This study added to evidence that walking speed may be important to consider when assessing the risk of cognitive impairment and impaired cognitive domain functions in older adults, and we can speculate that exercise intervention may serve as a means of primary prevention of cognitive decline. Future studies should explore a causal relationship between walking speed and cognitive impairment and impaired cognitive domain functions.

## Data availability statement

The original contributions presented in the study are included in the article/supplementary material, further inquiries can be directed to the corresponding author/s.

## Ethics statement

The study protocol was approved by the Ethics Committee of the Shanghai University of Medicine and Health Sciences (2020-E4-6200-20-201032-03-210302197009090947). The patients/participants provided their written informed consent to participate in this study.

## Author contributions

HW and HZ performed the literature search, conceived and designed the study, analyzed the data, and drafted the manuscript. PC analyzed the data and critically revised the manuscript. YC, CG, and MC performed the literature search. All authors have read and approved the final version of the manuscript, and agree with the order of presentation of the authors.

## Funding

This research was supported by the grant of the Shanghai Sports Science and Technology Project (20Q005).

## Conflict of interest

The authors declare that the research was conducted in the absence of any commercial or financial relationships that could be construed as a potential conflict of interest.

## Publisher's note

All claims expressed in this article are solely those of the authors and do not necessarily represent those of their affiliated organizations, or those of the publisher, the editors and the reviewers. Any product that may be evaluated in this article, or claim that may be made by its manufacturer, is not guaranteed or endorsed by the publisher.

## Abbreviations

MMSE: Mini-Mental State Examination
OR: odds ratio
CI: confidence interval
MCI: mild cognitive impairment
IPAQ-SF: International Physical Activity Questionnaire Short Form
BMI: body mass index.

## References

[B1] Bohannon R. W. Wang Y. C. (2019). Four-meter gait speed: normative values and reliability determined for adults participating in the NIH toolbox study. Arch. Phys. Med. Rehabil. 100, 509–513. 10.1016/j.Apmr.2018.06.031 30092204 PMC6363908

[B2] Chou M. Y. Nishita Y. Nakagawa T. Tange C. Tomida M. Shimokata H.. (2019). Role of gait speed and grip strength in predicting 10-year cognitive decline among community-dwelling older people. BMC Geriatr. 19, 186. 10.1186/S12877-019-1199-7 31277579 PMC6612180

[B3] Coelho-Junior H. J. Uchida M. C. Goncalves I. O. Calvani R. Rodrigues B. Picca A.. (2021). Age- and gender-related changes in physical function in community-dwelling brazilian adults aged 50 to 102 years. J. Geriatr. Phys. Ther. 44, E123–E131. 10.1519/JPT.0000000000000246 31693536

[B4] Giri M. Chen T. Yu W. Lu Y. (2016). Prevalence and correlates of cognitive impairment and depression among elderly people in the world's fastest growing city, Chongqing, People's Republic of China. Clin. Interv. Aging. 11, 1091–8. 10.2147/CIA.S113668 27574409 PMC4990376

[B5] Grande G. Vetrano D. L. Fratiglioni L. Marseglia A. Vanacore N. Laukka E. J.. (2020). Disability trajectories and mortality in older adults with different cognitive and physical profiles. Aging. Clin. Exp. Res. 32, 1007–1016. 10.1007/S40520-019-01297-1 31471890 PMC7260142

[B6] Hackett R. A. Davies-Kershaw H. Cadar D. Orrell M. Steptoe A. (2018). Walking speed, cognitive function, and dementia risk in the English longitudinal study of ageing. J Am Geriatr Soc. 66, 1670–1675. 10.1111/jgs.15312 29508385 PMC6127007

[B7] Hooghiemstra A. M. Ramakers I. Sistermans N. Pijnenburg Ya L Aalten P. Hamel R. E. G. (2017). Gait speed and grip strength reflect cognitive impairment and are modestly related to incident cognitive decline in memory clinic patients with subjective cognitive decline and mild cognitive impairment: findings from the 4C study. J. Gerontol. A Biol. Sci. Med. Sci. 72, 846–854. 10.1093/Gerona/Glx003 28177065

[B8] Ke Y. Xu J. Zhang X. Guo Q. Zhu Y. (2021). Association between serum follicle-stimulating hormone and sarcopenia and physical disability among older chinese men: evidence from a cross-sectional study. Front. Med. (Lausanne) 8, 724649. 10.3389/Fmed.2021.724649 35059409 PMC8764298

[B9] Knapstad M. K. Steihaug O. M. Aaslund M. K. Nakling A. Naterstad I. F. Fladby T.. (2019). Reduced walking speed in subjective and mild cognitive impairment: a cross-sectional study. J. Geriatr. Phys. Ther. 42, E122–E128. 10.1519/JPT.0000000000000157 29298174

[B10] Kondo R. Miyano I. Lee S. Shimada H. Kitaoka H. (2021). Association between self-reported night sleep duration and cognitive function among older adults with intact global cognition. Int. J. Geriatr. Psychiatry. 36, 766–774. 10.1002/gps.5476 33219536

[B11] Lavedan A. Viladrosa M. Jurschik P. Botigue T. Nuin C. Masot O.. (2018). Fear of falling in community-dwelling older adults: a cause of falls, a consequence, or both?. PLoS ONE. 13, E0194967. 10.1371/Journal.Pone.0194967 29596521 PMC5875785

[B12] Lee S. Kim E. Y. Shin C. (2019). Longitudinal association between brain volume change and gait speed in a general population. Exp. Gerontol. 118, 26–30. 10.1016/j.Exger.2019.01.004 30611726

[B13] Liu L. Qian X. Chen Z. He T. (2020). Health literacy and its effect on chronic disease prevention: evidence from China's data. BMC Public Health. 20, 690. 10.1186/S12889-020-08804-4 32410604 PMC7227325

[B14] Liu Y. Ma W. Li M. Han P. Cai M. Wang F.. (2021). Relationship between physical performance and mild cognitive impairment in chinese community-dwelling older adults. Clin. Interv. Aging. 16, 119–127. 10.2147/CIA.S288164 33469279 PMC7811437

[B15] Morley J. E.. (2018). An Overview of Cognitive Impairment. Clin. Geriatr. Med. 34, 505–513. 10.1016/j.Cger.2018.06.003 30336985

[B16] Peer M. Salomon R. Goldberg I. Blanke O. Arzy S. (2015). Brain system for mental orientation in space, time, and person. Proc. Natl. Acad. Sci. U.S.A. 112, 11072–7. 10.1073/Pnas.1504242112 26283353 PMC4568229

[B17] Rong J. Chen G. Wang X. Ge Y. Meng N. Xie T.. (2019). Correlation between depressive symptoms and quality of life, and associated factors for depressive symptoms among rural elderly in Anhui, China. Clin. Interv. Aging. 14, 1901–1910. 10.2147/CIA.S225141 31806946 PMC6839580

[B18] Rosenberg A. Ngandu T. Rusanen M. Antikainen R. Backman L. Havulinna S.. (2018). Multidomain lifestyle intervention benefits a large elderly population at risk for cognitive decline and dementia regardless of baseline characteristics: the FINGER trial. Alzheimers Dement 14, 263–270. 10.1016/j.Jalz.2017.09.006 29055814

[B19] Rosso A. L. Verghese J. Metti A. L. Boudreau R. M. Aizenstein H. J. Kritchevsky S.. (2017). Slowing gait and risk for cognitive impairment: the hippocampus as a shared neural substrate. Neurology 89, 336–342. 10.1212/WNL.0000000000004153 28659421 PMC5574674

[B20] Saez De Asteasu M. L. Martinez-Velilla N. Zambom-Ferraresi F. Casas-Herrero A. Cadore E. L. Galbete A.. (2019). Assessing the impact of physical exercise on cognitive function in older medical patients during acute hospitalization: secondary analysis of a randomized trial. PLoS Med. 16, E1002852. 10.1371/Journal.Pmed.1002852 PMC6611563 31276501

[B21] Sanders L. M. J. Hortobagyi T. Karssemeijer E. G. A. Van Der Zee E. A. Scherder E. J. A. Van Heuvelen M. J. G.. (2020). Effects of low- and high-intensity physical exercise on physical and cognitive function in older persons with dementia: a randomized controlled trial. Alzheimers Res. Ther. 12, 28. 10.1186/S13195-020-00597-3 PMC7082953 32192537

[B22] Schrack J. A. Zipunnikov V. Simonsick E. M. Studenski S. Ferrucci L. (2016). Rising energetic cost of walking predicts gait speed decline with aging. J. Gerontol. A Biol. Sci. Med. Sci. 71, 947–53. 10.1093/Gerona/Glw002 26850913 PMC4906328

[B23] Su L. Hayes L. Soteriades S. Williams G. Brain Sa E, Firbank, M. J.. (2018). Hippocampal stratum radiatum, lacunosum, and moleculare sparing in mild cognitive impairment. J. Alzheimers Dis. 61, 415–424. 10.3233/JAD-170344 29171994 PMC5819729

[B24] Tirkkonen A. Kulmala J. Hanninen T. Tormakangas T. Stigsdotter Neely A. Sipila S.. (2021). Associations between physical and executive functions among community-dwelling older men and women. J. Aging Phys. Act. 30, 332–339. 10.1123/Japa.2021-0075 34453020

[B25] Todd T. P. Bucci D. J. (2015). Retrosplenial cortex and long-term memory: molecules to behavior. Neural. Plast. 2015, 414173. 10.1155/2015/414173 PMC4562169 26380115

[B26] Toots A. T. M. Taylor M. E. Lord S. R. Close J. C. T. (2019a). Associations between gait speed and cognitive domains in older people with cognitive impairment. J. Alzheimers Dis. 71, S15–S21. 31256121 10.3233/JAD-181173

[B27] Toots A. T. M. Taylor M. E. Lord S. R. Close J. C. T. (2019b). Associations between gait speed and cognitive domains in older people with cognitive impairment. J. Alzheimers Dis. 71, S15–s2. 10.3233/JAD-181173 31256121

[B28] Wu J. Song X. Chen G. C. Neelakantan N. Van Dam R. M. Feng L.. (2019). Dietary pattern in midlife and cognitive impairment in late life: a prospective study in Chinese adults. Am. J. Clin. Nutr. 110, 912–920. 10.1093/Ajcn/Nqz150 31374567 PMC6766457

[B29] Xie H. Mayo N. Koski L. (2011). Predictors of future cognitive decline in persons with mild cognitive impairment. Dement Geriatr. Cogn. Disord. 32, 308–17. 10.1159/000334996 22286544

[B30] Yamawaki N. Radulovic J. Shepherd G. M. A. (2016). Corticocortical Circuit Directly Links Retrosplenial Cortex to M2 in the Mouse. J. Neurosci. 36, 9365–74. 10.1523/JNEUROSCI.1099-16.2016 27605612 PMC5013186

[B31] Zhang H. Ma W. Chen Y. Wang F. Wang J. Han P.. (2021). Long sleep duration associated with cognitive impairment in chinese community-dwelling older adults. J. Nerv. Ment. Dis. 209, 925–932. 10.1097/NMD.0000000000001401 34333503

[B32] Zhang M. Liu T. Li C. Wang J. Wu D. (2019). Physical performance and cognitive functioning among individuals with diabetes: findings from the China health and retirement longitudinal study baseline survey. J. Adv. Nurs. 75, 1029–1041. 10.1111/jan.13901 30397937

[B33] Zuo M. Gan C. Liu T. Tang J. Dai J. Hu X.. (2019). Physical predictors of cognitive function in individuals with hypertension: evidence from the CHARLS basline survey. West J Nurs Res, 41, 592–614. 10.1177/0193945918770794 29742988

